# Anti-Infective Screening of Selected Nine Cannabinoids Against *Clostridium perfringens* and Influenza A (H5N1) Neuraminidases, and SARS-CoV-2 Main Protease and Spike Protein Interactions

**DOI:** 10.3390/cimb47030185

**Published:** 2025-03-12

**Authors:** Thanet Pitakbut, Oliver Kayser

**Affiliations:** 1 Pharmaceutical Biology, Department of Biology, Friedrich-Alexander-Universität Erlangen-Nürnberg (FAU), 91058 Erlangen, Germany; 2 Technical Biochemistry, Department of Biochemical and Chemical Engineering, TU Dortmund University, 44227 Dortmund, Germany

**Keywords:** cannabinoids derivatives, anti-infective properties, *Clostridium perfringens* neuraminidase, influenza A (H5N1) neuraminidase, SARS-CoV-2 main protease, SARS-CoV-2 spike protein–human ACE2 interaction

## Abstract

Recently, cannabinoids have gained scientific interest as a promising anti-infective natural product class, as reported in several studies. However, the existing knowledge is mainly limited to common cannabinoids like THC and CBD. Therefore, this study aims to fill the knowledge gap by investigating the anti-infective potential of nine selected cannabinoids (both common and rare cannabinoids): THC, CBD, CBC, CBE, CBF, CBG, CBL, CBN, and CBT against *Clostridium perfringens* and Influenza A (H5N1) neuraminidases and SARS-CoV-2 main protease and spike protein–human ACE2 interaction using a standard in vitro biochemical enzyme-binding assay. As a result, to the authors’ knowledge, this study is the first to demonstrate the most promising effect of CBG over others in its class against *C. perfringens* and influenza A (H5N1) neuraminidases and SARS-CoV-2 main protease and spike protein–human ACE2 interaction. In comparison to CBG, CBD and THC were the second and third most promising candidates. Meanwhile, the other derivatives, such as CBC, CBE, CBF, CBL, CBN, and CBT, showed at least one anti-infective effect. Our findings during the early drug discovery process indicate a promising anti-infective potential of cannabinoids, which can be considered for further investigation in a biological setup.

## 1. Introduction

Cannabinoids are known as secondary plant metabolites derived from *Cannabis sativa* L. [1]. For centuries, humans have utilized cannabis plants for multiple purposes. Like in Asia, cannabis has been used as cuisine for cooking traditional meals [2,3] due to its unique taste, flavor [4,5], and appetite-stimulating effect [6]. However, after discovering tetrahydrocannabinol or THC (a psychoactive substance), using cannabis plants was legally prohibited [7]. Recently, many countries worldwide have started legalizing cannabis usage after scientifically discovering cannabis and cannabinoids’ health benefits [7]. Additionally, since the COVID-19 pandemic, cannabinoids, like cannabidiol (CBD), cannabigerol (CBG), and cannabicyclol (CBL), have been investigated and demonstrated promising inhibitory activity against the SARS-CoV-2 virus [8,9]. This has promoted popularity in cannabinoid research. According to current knowledge, there are more than ten classes of plant-based cannabinoids [10]. However, only major cannabinoid classes like THC, CBD, CBG, and CBL are usually investigated. Meanwhile, the other minor cannabinoids are excluded [11], which is a current knowledge gap.

Therefore, the potential antimicrobial landscape of these cannabinoids remains unknown. To close this knowledge gap, the authors aim to explore cannabinoids’ anti-infective properties of nine cannabinoid classes covering both major cannabinoids like THC, CBD, CBG, and CBL and minor cannabinoids such as cannabichromene (CBC), cannabielsoin (CBE), cannabifuran (CBF), cannabinol (CBN), and cannabitriol (CBT) against *Clostridium perfringens* and influenza A (H5N1) neuraminidases and SARS-CoV-2 main protease and spike protein–human ACE2 interaction.

Previously, the authors found that THC and CBD demonstrated a firm effect against SARS-CoV-2 main protease activity in an enzyme-binding assay [8]. Later, in a cell-based experiment, the authors discovered that other derivatives like CBG and CBL could block viral entry and inhibit SARS-CoV-2 reproduction [9]. Our previous findings here are in line with other reports [12,13,14]. One pronounced study by Nguyen and colleagues showed that patients who took 100 mg/mL of CBD oral solution demonstrated a significant reduction in the risk of SARS-CoV-2 infection compared to patients who did not take CBD solution in a cohort study [14]. At the same time, CBD’s antibacterial properties were unfolded, and one significant finding was an inhibitory activity against antimicrobial-resistant bacteria like methicillin-resistant *Staphylococcus aureus* (MRSA) in both in vitro and in vivo models [15,16,17]. Furthermore, there were also multiple reports on cannabinoids’ anti-influenza effects. However, these studies primarily focused on an inflammatory response via cannabinoids’ receptors [18,19,20], not a direct antiviral effect like inhibiting the viral replication process.

Generally, cannabinoids can be divided into two major groups based on their origins. The first is synthetic or unnatural cannabinoids, and the second is natural cannabinoids [21,22]. Natural cannabinoids can be further categorized into two sub-groups. The first sub-group is endocannabinoids (produced inside animals, including humans). The second is phytocannabinoids (biosynthesized in plants) [21,22]. In this study, cannabinoids represent only plant-based cannabinoids. Typically, common cannabinoids refer to major cannabinoids produced by plants in great quantity, such as THC and CBD, while rare cannabinoids refer to minor cannabinoids with less quantity. Based on current knowledge, there are at least ten classes of cannabinoids. However, most studies only investigate common cannabinoids‚ and often exclude rare cannabinoids, as mentioned in the paragraph above [23,24].

**Figure 1:**
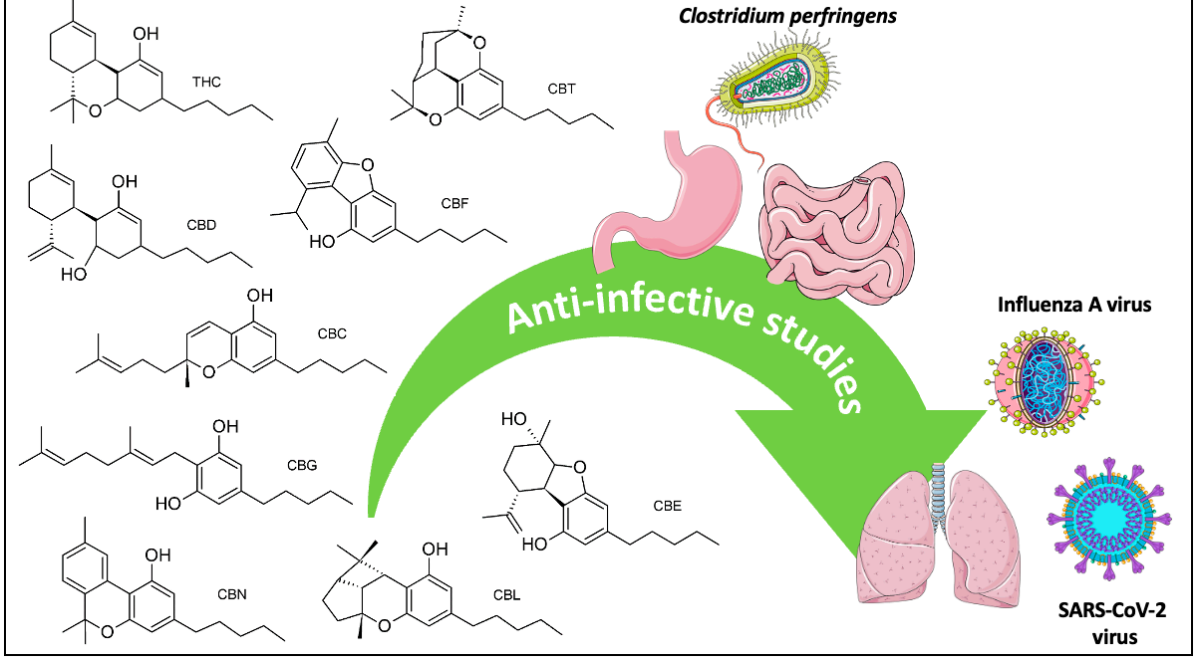
Chemical structures of cannabinoid derivatives and research study design. THC = tetrahydrocannabinol, CBC = cannabichromene, CBD = cannabidiol, CBE = cannabielsoin, CBF = cannabifuran, CBG = cannabigerol, CBL = cannabicyclol, CBN = cannabinol, CBT = cannabitriol. (The figure was partly generated using Servier Medical Art, provided by Servier, licensed under a Creative Commons Attribution 3.0 unported license).

To address this issue, the authors selected nine different cannabinoid classes covering both common cannabinoids (THC, CBD, CBG, and CBL) and rare cannabinoids (CBC, CBE, CBF, CBN, and CBT), as shown in Figure 1, and investigated their anti-infective mechanisms against three drug targets. The first target is neuraminidase, and two different neuraminidase enzymes are used in this study. One is from *C. perfringens*, and another one is from influenza A virus (IAV) H5N1 (A/Anhui/1/2005). The second target is the SARS-CoV-2 main protease, and the third is the SARS-CoV-2 spike protein to evaluate an inhibitory effect of cannabinoids against viral spike protein–human ACE2 interaction. According to the authors’ best knowledge, this study is the first to report a comprehensive analysis of nine classes of cannabinoids against these proteins.

## 2. Materials and Methods

### 2.1. Chemicals

The authors purchased CBD and THC (purity > 98%) from Cayman Chemical Company (Ann Arbor, MI, USA). In contrast, other cannabinoid derivatives such as CBC, CBE, CBF, CBG, CBL, CBN, and CBT (high purity up to 98%) were synthesized by the authors’ colleague, Gia-Nam Nguyen, an organic chemist who worked in the same laboratory as the authors. All essential information regarding cannabinoids’ synthesis and characterization (chromatographic and spectroscopic analyses) can be found in Gia-Nam Nguyen and the team’s previous report [25].

### 2.2. Anti-C. perfringens Neuraminidase Assay

By modifying previously reported protocols, the authors established an in-house *C. perfringens* inhibitory neuraminidase assay [26,27,28]. Since no clinical reference (standard drug) explicitly designed for bacterial neuraminidase is available [29], the authors used a non-clinical inhibitor, quercetin, as a non-clinical positive control, as used in the previous report [30].

In brief, a 96-well plate was used in this assay, and the total volume was 200 µL. First, 50 µL of 50 mM sodium acetate pH 5.5 was added into each well. Second, 50 µL of 200 µg/mL sample or positive control (quercetin, Sigma-Aldrich, Munich, Germany) solution was added next. For negative control, the author used a 4% DMSO solution (1% DMSO at the final concentration). Third, the authors added 50 µL of 50 µU/mL *C. perfringens* neuraminidase (Sigma-Aldrich) and thoroughly mixed it with the previous solution. Then, 15 min of incubation took place at 37 degrees. After the incubation, at the final step, the authors added 50 µL of 80 µM 4MUNANA (2′-(4-Methylumbelliferyl)-α-D-N-acetylneuraminic acid sodium salt hydrate, Sigma-Aldrich, St. Louis, MO, USA) into the assay solution and monitored the reaction for 10 min. The authors used the FLUOstar^®^ Omega microplate reader (BMG Labtech, Ortenberg, Germany) to monitor the reaction via fluorescence signal, and λ excitation was set at 368 nm while λ emission was set as 460 nm.

The authors calculate percent (%) inhibition of test sample and positive control by comparing the obtained signals to negative control using the first Equation (1) shown below.

(1) $$\%\;inhibition=\frac{\left({FI}_{negative}-{FI}_{sample/positive}\right)}{{FI}_{negative}}\times 100$$

FI on the equation represented fluorescence signal intensity read by the FLUOstar^®^ Omega microplate reader. All samples were tested in triplicates (*n* = 3), and the % inhibition was reported in $\bar{x}$ ± SD.

Only the samples that exhibited % inhibition of more than 80% were considered positive hit and further subject to evaluate their IC_50_ values (the concentration inhibiting 50% enzymatic reaction) via curve-fitting method using the second Equation (2) shown below as a model.

(2) $$\%\;inhibition=\frac{1}{\left(1-({\frac{{IC}_{50}}{concentration})}^{Hill}\right)}$$

In the model (second equation), % inhibition refers to an inhibitory activity caused by a particular concentration, and hill represents the hill coefficient value. The authors used five concentration of each positive hit to determine its IC_50_ value using both Microsoft (MS) Excel and RStudio program (version 4.2.2).

### 2.3. Anti-Influenza A Viral Neuraminidase Assay

A similar assay protocol as above was applied to evaluate the anti-influenza A viral neuraminidase. In this assay, influenza A viral (H5N1), H255Y mutated neuraminidase obtained from SinoBiological (Beijing, China) was used. The viral neuraminidase concentration was adjusted to 1 mU/mL. Since there is a standard influenza A neuraminidase drug available, the authors used zanamivir (clinical reference) obtained from Sigma-Aldrich as a positive control used in the previous reports [31,32]. Finally, the authors used the same first (1) and second (2) equations to calculate cannabinoids’ % inhibition and IC_50_ values against viral neuraminidase.

### 2.4. Anti-SARS-CoV-2 Main Protease Activity Assay

Unlike neuraminidase assays, the authors evaluated anti-SARS-CoV-2 main protease effect using a ready-to-use assay kit purchased from BPS Biosciences (San Diego, CA, USA). As mentioned in the authors’ previous study, the authors conducted this experiment following a company manufacturing guideline strictly [8]. In short, the authors prepared a proper concentration of SARS-CoV-2 main protease as suggested in the guideline. Next, the authors mixed 30 µL of prepared SARS-CoV-2 main protease solution with 10 µL of 500 µg/mL sample solution dissolved in 5% DMSO or 500 µM GC376 (positive control, provided with the assay kit). However, for the negative control, the authors used a 5% DMSO solution (1% DMSO at the final concentration). Next, the authors incubated the mixed solution for 30 min. Finally, the authors added 10 µL o10 µM substrate solution, starting a reaction. The same microplate reader in previous experiments was used to monitor the enzymatic response every hour for four hours. Finally, both mentioned equations as above were applied here to evaluate % inhibition and IC_50_ values in this experiment.

### 2.5. Anti-SARS-CoV-2 Spike Protein–Human ACE2 Interaction Assay

Similar to anti-SAR-CoV-2 main protease assay, the authors evaluated the anti-SARS-CoV-2 spike protein–human ACE2 interaction activity of cannabinoid derivatives using a ready-to-use TR-FRET assay kit purchased from BPS Biosciences (San Diego, CA, USA). The authors performed this assay following a manufacturer’s guideline strictly. In short, the authors added 5 µL of 12 nM ACE2 labeled with europium (Eu) dye into each well. Later, the authors mixed 5 µL of 400 µg/mL test solution dissolved in 4% DMSO (Final concentration of 100 µg/mL in 1% DMSO) or 4% DMSO (negative control) into the well. However, no positive control came with the purchased kit, and no clinical reference or standard drug for the assay was available. Following previous reports, the authors used 5 µL of 400 µg/mL of pinostrobin solution (similar concentration as above) as a non-clinical positive control [33,34]. Later, another 5 µL of a dyed-labeled acceptor solution was added. Finally, the authors pipetted 5 µL of 200 nM spike S1 protein labeled with Biotin and added it into the wells. The same microplate reader, FLUOstar^®^ Omega, read the reaction after an hour of incubation. The authors set two different channels on the microplate reader to obtain the TR-FRET signal, as suggested in the protocol. For the first channel, the authors used 340 nm for λ excitation, 620 nm for λ emission, 10 µs for a lag time, and 500 µs for an integration time. For the second channel, the authors applied nearly the same parameter values, except λ emission, which was changed to 665 nm. The first equation was used to calculate the % inhibition of all samples tested in this experiment.

### 2.6. Statistical Analysis

In the screening step, the authors performed descriptive statistical analysis through Microsoft (MS) Excel 365 to obtain % inhibition of each cannabinoid in the form of $\bar{x}$ and SD from the first equation [8]. Next, a two-step analysis was performed to obtain an IC_50_ value of each positive hit (inhibition > 80%). First, the authors obtained preliminary IC_50_ and Hill coefficient values using a solver package from MS Excel 365 after performing curve-fitting using the second equation as a model [35,36]. Later, the authors used the preliminary IC_50_ and Hill coefficient values from MS Excel for fine-tuning and obtained more comprehensive statistic parameters such as SE (standard error of mean), 95% confidence intervals, and R^2^ values via the stat package from the RStudio program (version 4.2.2) [37,38,39,40]. Finally, the authors generated all graphs and heatmaps through MS Excel.

## 3. Results

### 3.1. Anti-C. perfringens Neuraminidase Activity

The authors screened anti-*C. perfringens* neuraminidase activity of seven cannabinoids, such as THC, CBC, CBD, CBG, CBL, CBN, and CBT, at 50 µg/mL. The obtained % inhibitory activity of each cannabinoid was compared to quercetin, a non-clinical positive control. Based on the previous reports, quercetin exhibited a potent inhibitory effect against *C. perfringens* neuraminidase [41,42]. There are two main reasons why the authors did not select clinical references here. The first is that standard viral neuraminidase inhibitor drugs like oseltamivir and zanamivir do not effectively inhibit bacterial neuraminidase [41,42], and the second is that there is currently no clinical drug for bacterial neuraminidase available [29]. Therefore, the author decided to use quercetin as a positive control.

**Figure 2:**
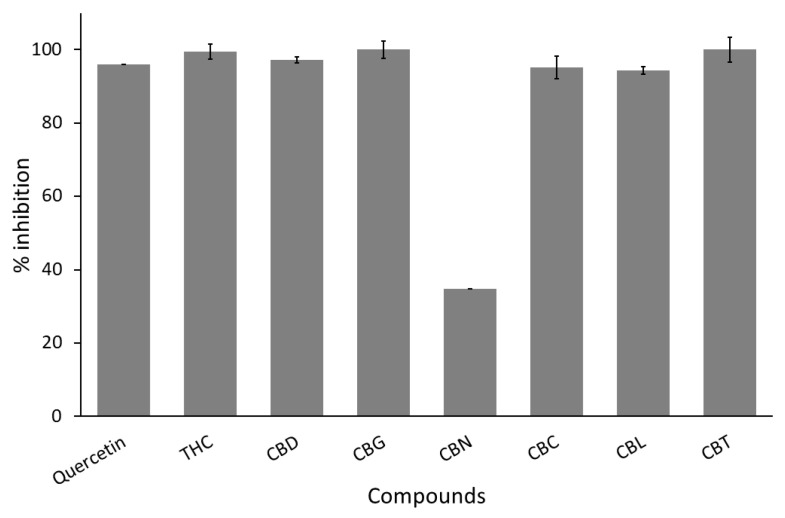
Screening anti-*C. perfringens* neuraminidase of cannabinoid derivatives, namely, THC, CBD, CBG, CBN, CBC, CBL, and CBT, compared to quercetin as a positive control. All test samples are tested at a concentration of 50 µg/mL as a final concentration. Each compound is tested in triplicates (*n* = 3).

Figure 2 exhibited results obtained from the authors’ screening, showing that nearly all cannabinoids inhibited *C. perfringens* neuraminidase firmly, except CBN. The authors used % inhibition of more than 80% as a selection criterion and defined any cannabinoid passing this criterion as a positive hit. Therefore, six cannabinoids, such as THC, CBD, CBG, CBC, CBL, and CBT, were identified as hits and were subjected to determine their inhibition potency, IC_50_ values, a concentration that inhibited 50% enzymatic activity. The % inhibition of each cannabinoid was shown in Table 1.

**Figure 3:**
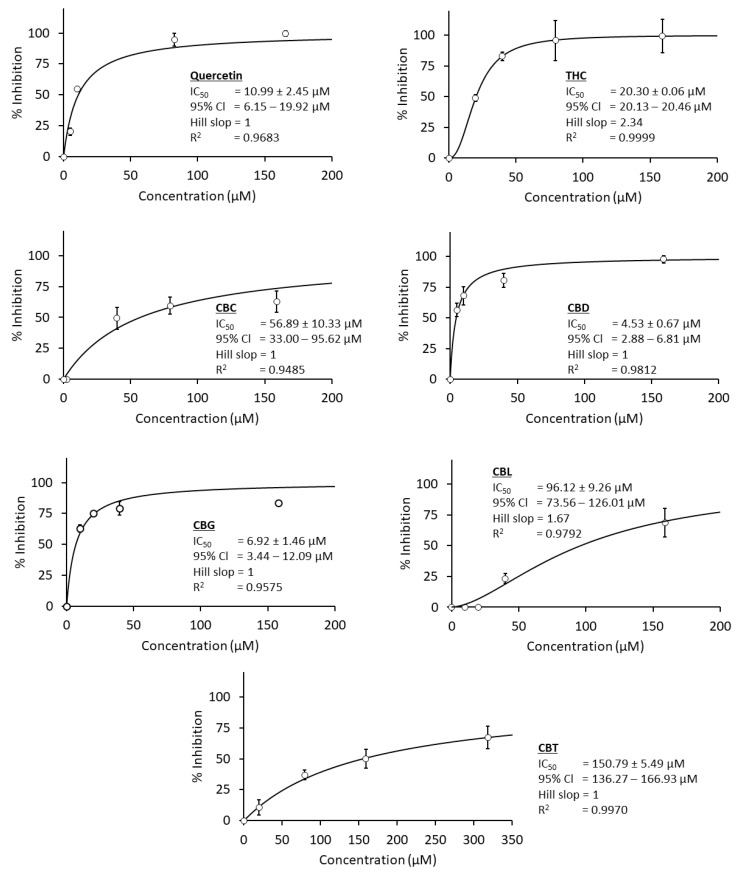
A comparison of the concentration–response curve of each selected cannabinoid and quercetin as a positive control. At least five concentrations of each compound are used to calculate IC_50_ values via a nonlinear regression model (Equation (2)), and each concentration is tested in triplicates (*n* = 3).

In Figure 3, the result revealed that the IC_50_ values of two cannabinoids, such as CBD and CBG, were lower than the IC_50_ value of quercetin (a non-clinical positive control), indicating a more potent inhibitory activity. For example, the IC_50_ values of CBD and CBG at 4.5 µM and 6.9 µM were approximately 2.5 to 1.5 folds lower than the IC_50_ value of quercetin, around 11 µM. In contrast, the other cannabinoids like THC, CBC, CBL, and CBT exhibited less potency than the positive control (quercetin), nearly two-fold, five-fold, and more, accordingly. Their IC_50_ values were approximately 20.3 µM, 56.9 µM, 96.1 µM, and 150.8 µM. Notably, the standard deviation (SD) of the last two highest concentrations of THC was more significant than the lower concentrations. It was due to a poor solubility of THC, which was also found in the authors’ previous report [8]. A summary of each cannabinoid IC_50_ value against *C. perfringens* neuraminidase was provided in Table 2.

### 3.2. Anti-Influenza A Virus Neuraminidase Activity

After cannabinoids exhibited promising inhibitory activity against bacterial (*C. perfringens*) neuraminidase in a prior experiment, the authors further evaluated the anti-neuraminidase activity against the influenza A virus (IAV). Additionally, the authors extended our screening by adding two more cannabinoid derivatives, CBE and CBF. Finally, the author used zanamivir (an anti-influenza drug) as a clinical positive control in this experiment. The authors did not select oseltamivir as a clinical reference due to a mutation at the H255Y position on the enzyme. This mutation contributed to oseltamivir resistance, as reported by the WHO [43].

**Figure 4:**
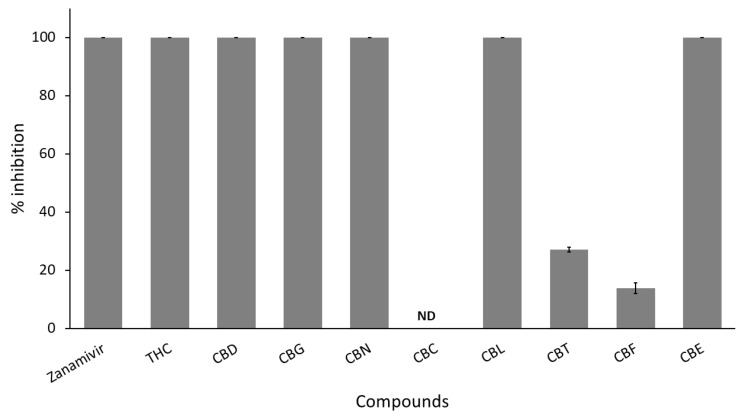
Screening anti-IAV neuraminidase of nine cannabinoid derivatives (THC, CBD, CBG, CBN, CBC, CBL, CBT, CBF, and CBE) compared to zanamivir as a clinical positive control. All test samples are tested at a concentration of 50 µg/mL as a final concentration. Each compound is tested in triplicates (*n* = 3). ND represents “non-detectable”, indicating no inhibitory activity against IVA neuraminidase.

Here, the authors studied nine cannabinoid derivatives. Figure 4 shows six active cannabinoids, THC, CBD, CBE, CBG, CBL, and CBN, completely inhibiting IAV neuraminidase activity (100% inhibition). On the contrary, CBC showed no inhibitory activity against IAV neuraminidase. At the same time, CBT and CBF showed only 10% to 20% inhibition against viral neuraminidase, as shown in Table 1. Therefore, only six cannabinoids were considered positive hits and subjected to evaluate their inhibitory potency, IC_50_ values.

**Figure 5:**
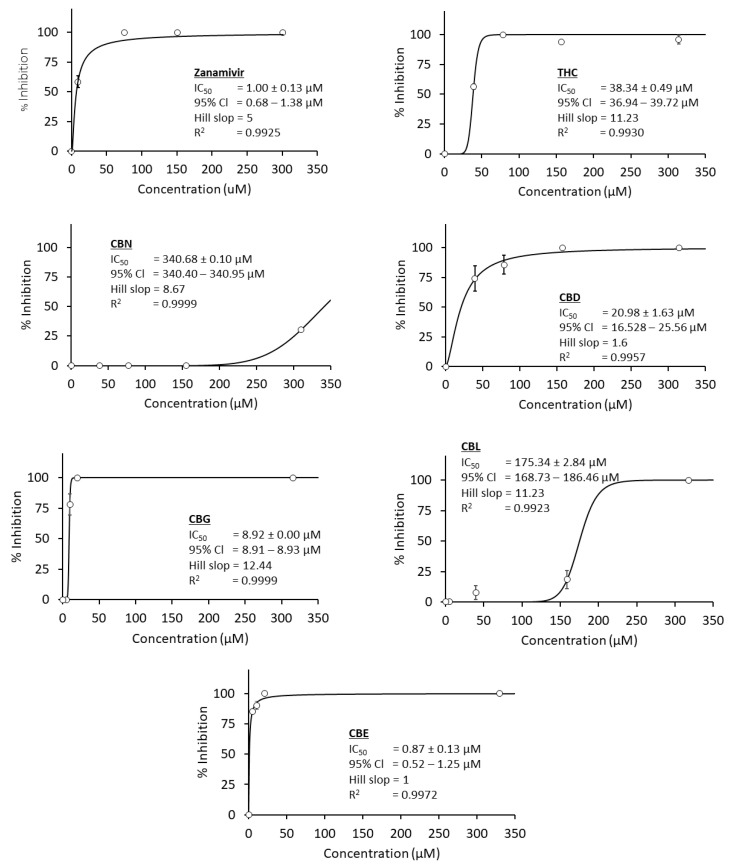
A comparison of the concentration–response curve of six selected cannabinoids and zanamivir as a positive control. Five concentrations of each compound are used to calculate IC_50_ values using the curve-fitting method via Equation (2), and each concentration is tested in triplicates (*n* = 3).

Figure 5 showed that CBE was the only cannabinoid exhibiting a similar inhibitory potency (IC_50_ value = 0.8 µM) as zanamivir, a clinical positive control and clinical reference (IC_50_ value = 1.0 µM). Meanwhile, CBG was the second-strongest potency with IC_50_ value = 8.9 µM. On the other hand, the other cannabinoid derivatives exhibited moderate to weak inhibitory activity against IAV neuraminidase. For example, IC_50_ values of CBD and THC were 20.9 µM and 38.3 µM, while CBL and CBN showed IC_50_ values of 175.3 µM and 340.7 µM. Finally, Table 2 exhibited a comprehensive overview of each cannabinoid IC_50_ value against IVA neuraminidase.

### 3.3. Anti-SARS-CoV-2 Activities

#### 3.3.1. Anti-SARS-CoV-2 Main Protease

In the authors’ previous report, three cannabinoids (THC, CBD, and CBN) were evaluated for the anti-SARS-CoV-2 main protease activity, and only THC and CBD demonstrated a potent anti-SARS-CoV-2 main protease activity (IC_50_ values = 1.9 µM and 16.2 µM, respectively) [8]. Therefore, in this study, the authors evaluated six other cannabinoid derivatives, such as CBC, CBE, CBF, CBG, CBL, and CBT, against the SARS-CoV-2 main protease.

**Figure 6:**
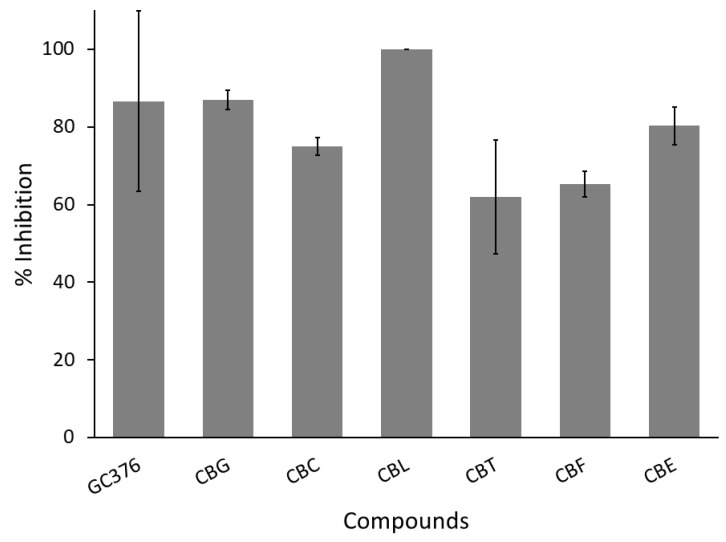
Screening anti-SARS-CoV-2 main protease of six cannabinoid derivatives (CBG, CBC, CBL, CBT, CBF, and CBE) compared to GC376 as a positive control. All test samples are tested at a concentration of 100 µg/mL as a final concentration. Each compound is tested in triplicates (*n* = 3).

In this experiment, the author selected a ready-to-use assay kit from BPS Biosciences to evaluate cannabinoids’ anti-SARS-CoV-2 main protease activity, as mentioned in Section 2. The assay kit came with a positive control, GC376 (a viral protease inhibitor drug candidate currently being developed by Anivive Lifesciences). Only two cannabinoids, CBG and CBL, showed more than 80% inhibitory activity, passing the selection criterion in the screening step (Figure 6 and Table 1). Therefore, these two positive hits were subjected to evaluate their IC_50_ values against the SARS-CoV-2 main protease.

**Figure 7:**
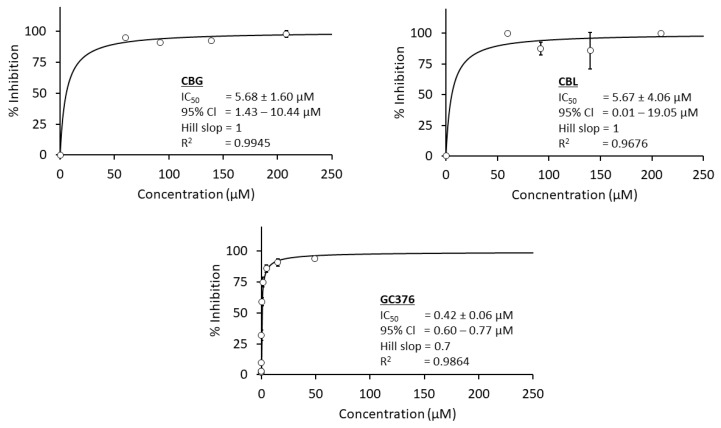
A comparison of the concentration–response curve of CBG, CBL, and GC376 (positive control) against SARS-CoV-2 main protease. Five concentrations of each cannabinoid are used to calculate IC_50_ values using the curve-fitting method via Equation (2), and each concentration is tested in triplicates (*n* = 3). The nonlinear regression analysis of GC376 is based on the company-provided data used in the authors’ previous report.

Figure 7 and Table 2 demonstrated the IC_50_ values of CBG and CBL at around 5.6 µM. Meanwhile, the IC_50_ value of GC376 (a positive control and antiviral main protease drug candidate) was 0.4 µM, lower than the positive hits. However, there was a notable limitation here. As shown in Figure 7, the tested lowest concentration of both CBG and CBL exhibited a high percent inhibition above 75%, and it was in the same activity range as the higher concentrations in a plateau or zero order phase. Therefore, the obtained value may not represent the true inhibitory potency of these cannabinoids. Ideally, the IC_50_ value must be re-evaluated for more realistic values. However, due to the limitation of CBG and CBL availability, the authors did not and decided to acquire the IC_50_ values heavily based on the mathematical equation supported by an enzyme-inhibitor interaction theory [44], which might be derived from actual values of CBG and CBL.

#### 3.3.2. Anti-SARS-CoV-2 Spike Protein–Human ACE2 Interaction

Based on the authors’ previous report, THC and CBD exhibited an inhibitory activity against human ACE2 catalytic activity [8]. Therefore, the authors hypothesize that cannabinoids might be able to interrupt the interaction between SARS-CoV-2 spike protein and human ACE2 even though recent studies have shown that inhibiting the human ACE2 catalytic activity could not block the SARS-CoV-2 spike protein–human ACE2 interaction effectively [45,46].

Like the anti-SARS-CoV-2 main protease assay, the authors purchased a ready-to-use assay kit from BPS Biosciences to perform this experiment. However, the assay kit here did not provide a positive control. Furthermore, no standard drug or clinical reference is available for blocking SARS-CoV-2 spike protein–human ACE2 interaction when authors perform this experiment. Therefore, the authors selected a non-clinical inhibitor as a positive control based on the literature and the availability of a compound. As a result, the authors selected pinostrobin as a non-clinical positive control in this experiment since there was support from previous studies [33,47,48] and it was available in the authors’ laboratory.

**Figure 8:**
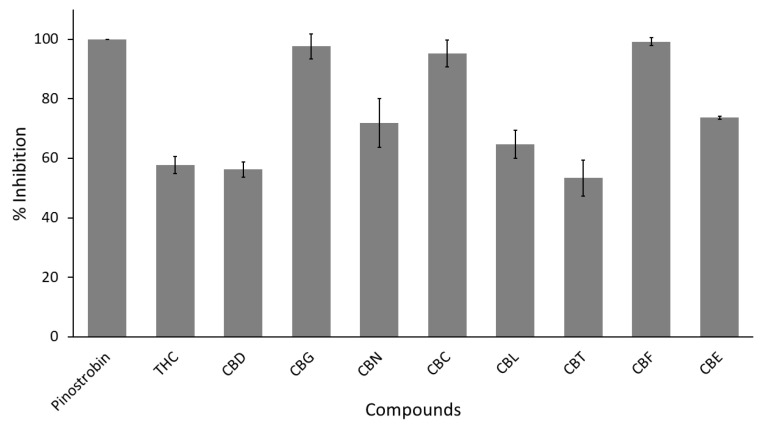
Screening activity of nine cannabinoid derivatives against SARS-CoV-2 spike protein–human ACE2 interaction. The screening concentration is 100 µg/mL. Pinostrobin is used as a positive control.

As expected, pinostrobin (a non-clinical positive control) completely inhibited interaction between SARS-CoV-2 spike protein and human ACE2 interaction (100% inhibition) in the screening step, Figure 8 and Table 1. On the other hand, only three cannabinoids, like CBG, CBC, and CBF, exhibited nearly 100% inhibition and were considered positive hits. Meanwhile, the other cannabinoids like THC, CBD, CBN, CBL, CBT, and CBE demonstrated an underperformance of less than 80% inhibition (Figure 8 and Table 1). Unlike all experiments above, the positive hits were not subjected to further determination of their IC_50_ value due to insufficient amounts.

Yet, the result here showed a proof-of-concept that some cannabinoids derivatives were able to inhibit the interaction between SARS-CoV-2 spike protein and human ACE2 receptor. Furthermore, our finding aligns with existing knowledge that a human ACE2 inhibitor (like THC and CBD [8]) cannot effectively block SARS-CoV-2 spike protein–human ACE2 interaction [45,46].

## 4. Discussion

Cannabinoids are unique bioactive molecules derived from cannabis plants (*Cannabis sativa* L.) [1]. Based on traditional knowledge, cannabis plants can be used as a cuisine [2,3], offering a distinctive taste and flavor with an appetite-stimulating effect [4,5,6]. However, after discovering a psychoactive cannabinoid, THC, using cannabis for any purpose was prohibited [7]. Therefore, our understanding regarding cannabinoids and cannabis plants is generally limited. However, recently, many countries worldwide have started to legalize cannabis and cannabinoids for research and medical uses [7], and modern science has revealed the health-promoting effects of cannabinoids [49,50]. By far, more than ten different classes of cannabinoids have been discovered [25]. However, usually, only common or major cannabinoids, like THC, CBD, CBG, and CBL, are under investigation [11], and this is a current knowledge gap.

In this study, the authors aim to fill this gap by screening selected nine cannabinoids covering both common (THC, CBD, CBG, and CBL) and rare cannabinoids (CBC, CBE, CBF, CBN, and CBT) against *C. perfringens* and IAV neuraminidases and SARS-CoV-2 main protease and spike protein–human ACE2 interaction using a standard in vitro colorimetric enzyme-binding assay. Our result in the early phase of drug discovery indicated a promising anti-infective potential of cannabinoids, and it is worth subjecting these cannabinoids for further investigation, validating cannabinoids’ anti-infective effect in cell-based and animal models.

**Figure 9:**
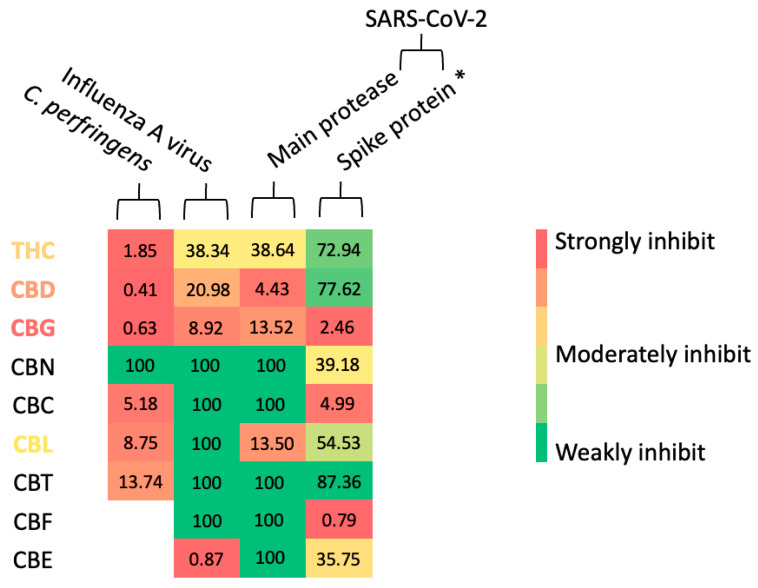
An overview of the anti-infective activities of cannabinoid derivatives found in this study is shown in a heatmap format. Asterisk (*) represents a calculation based on a result obtained from a screening study, while the other calculates based on IC_50_ values compared to a positive control or standard drug.

For a comprehensive discussion, the authors created a potency index (PI) for each assay by calculating a ratio between the IC_50_ value of cannabinoids of interest and the IC_50_ value of positive control used in each experiment. This PI value aimed to assist the authors in cross-comparing cannabinoids’ anti-infective potential, and the authors used PI values to generate a heatmap for comprehensive visualization (Figure 9). If the ratio between cannabinoids and positive control is equal or more than 100 times, the heatmap will show a green color, representing a weak inhibition. On the other hand, if the ratio between cannabinoids and positive control is close to 0, it will show a red color, representing a strong inhibitory effect on the heatmap. Finally, if the ratio is between 0 and 100, it will exhibit different shades of colors, from green to red, and consider a moderate effect. For more details, the authors provided relevant data for PI calculation in a Supplementary File.

### 4.1. Cannabinoids in Bacterial and Viral Neuraminidases

As shown earlier, the anti-influenza A viral neuraminidase activity of cannabinoids was tested. Compared to anti-bacterial *C. perfringens* neuraminidase, two extra cannabinoid derivatives, CBE and CBF, were exclusively included for the influenza A viral neuraminidase assay. Unlike the bacterial *C. perfringens* neuraminidase, in which nearly all tested cannabinoids were active, only four cannabinoids exhibited moderate to intense inhibitory activity in the antiviral counterpart, and five cannabinoids acted as weak inhibitors (Figure 4, Figure 5 and Figure 9). Even though the IC_50_ values of cannabinoids did not distinctively differ in both experiments, in the µM range (Table 2), the reason that fewer cannabinoids were categorized as potent inhibitors in the antiviral neuraminidase assay, shown in Figure 9, was due to a lower IC_50_ value of zanamivir (a clinical positive control and current anti-influenza drug). As explained earlier in this section, PI or potency index value was introduced to normalize the potency of tested cannabinoids with positive control used in each experiment. The authors used a clinical reference like zanamivir (IC_50_ = 1 µM) in the viral neuraminidase experiment, and it exhibited a lower IC_50_ value than a non-clinical reference like quercetin (IC_50_ = 11 µM) by 11 times. Therefore, this smaller IC_50_ value of zanamivir contributed to a more significant gap between the cannabinoids’ IC_50_ values and positive control ratio. Consequently, this wider gap led to fewer cannabinoids being listed in a potent category. Additionally, the author provided raw data by generating a heat map of Figure 9, which is in a Supplementary File for more details. Even if this is the case, three cannabinoids, such as THC, CBD, and CBG, exhibited promising activity with potent to moderate effects (PI values between 0.4 and 38) against bacterial and viral neuraminidases.

**Figure 10:**
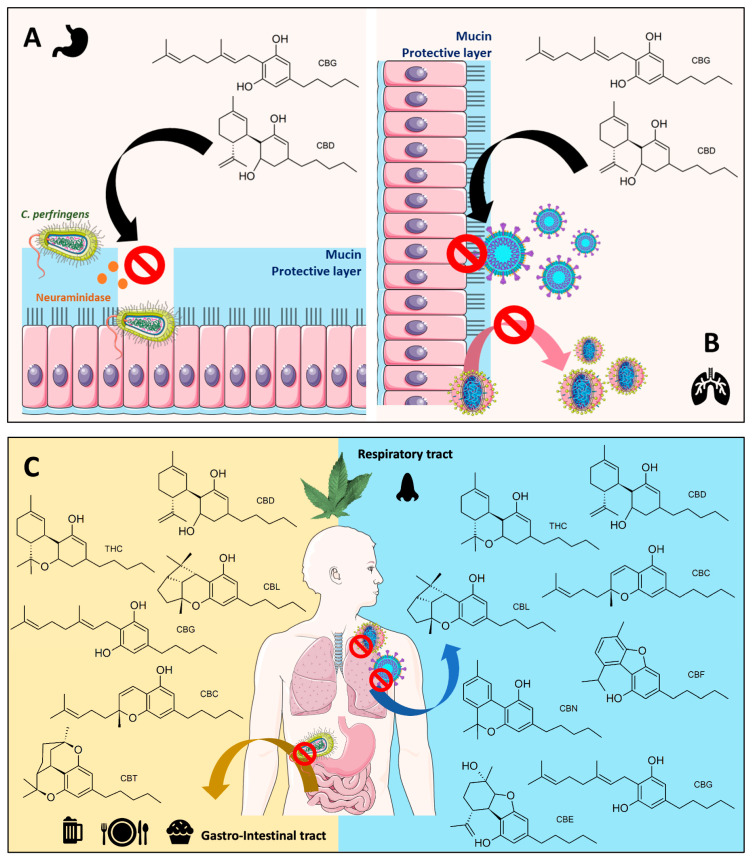
A summary of cannabinoids’ anti-infective mechanisms against gut–lung axis pathogens like *C. prefengens*, influenza A, and SARS-CoV-2 viruses. (**A**) Proposed cannabinoids’ antibacterial activity against *C. perfringens* via inhibiting neuraminidase. (**B**) Proposed cannabinoids’ antiviral mechanisms against influenza A and SARS-CoV-2 viruses involving mucin, a protective layer. (**C**) Potential lists of active cannabinoids affecting microbes in human gastro-intestinal and respiratory tract systems. (The figure was partly generated using Servier Medical Art, provided by Servier, licensed under a Creative Commons Attribution 3.0 unported license).

Interestingly, the human digestive and respiratory systems are protected by mucin [51] (Figure 10A,B). To initiate pathogenesis in the human digestive tract, bacteria like *C. perfringens* release a neuraminidase enzyme to destroy mucin and start colonization, as shown in Figure 10A. On the other hand, in the respiratory system, the influenza A virus uses neuraminidase in the late stage of viral replication, detaching newly reproduced viral cells from the human cellular hosts (Figure 10B) [52]. Therefore, inhibiting viral neuraminidase does not prevent viral colonization but diminishes viral reproduction, allowing the human immune response to eliminate the viruses more effectively [53]. Even though both enzymes exhibit a similar biochemical function, the protein sequences between bacteria and viruses are significantly different (only 16% similarity, Supplementary File). This structural diversity contributes to the lack of effectiveness of a viral neuraminidase inhibitor drug like zanamivir against bacterial neuraminidase [41,42]. Yet, THC, CBG, and CBN were able to inhibit both enzymatic activities in an in vitro setup. Our preliminary result here hints at the potential of these cannabinoids for further investigation and development. Since no current drug in the clinic targets bacterial and viral neuraminidase, developing a new inhibitor to inhibit both enzymes can be beneficial.

On the other hand, as presented in Figure 9, CBE exhibited a promising inhibitory activity toward influenza A viral neuraminidase, close to zanamivir (a standard drug), with a PI value of 0.87. This finding makes CBE an interesting candidate for further antiviral neuraminidase drug development in cell-based and in vivo studies. Additionally, the authors have not yet evaluated CBE’s antibacterial neuraminidase activity. Therefore, it is also essential to assess CBE activity against *C. perfringens* neuraminidase in the future.

### 4.2. Cannabinoids in SARS-CoV-2 Infection and Their Possible Correlation to Neuraminidase Inhibitions

In this study, the authors’ experiment revealed the potential anti-SARS-CoV-2 mechanisms of cannabinoids. The results showed that CBG and CBL exhibited dual anti-SARS-CoV-2 effects, inhibiting the main protease (essential enzyme for viral replication) and disrupting the interaction between the SARS-CoV-2 spike protein and human ACE2 receptor (vital viral entry mechanism). Even though both cannabinoids showed nearly identical potency against SARS-CoV-2 main protease with a PI value of around 13, CBG demonstrated a better activity against SARS-CoV-2 spike protein–human ACE2 interaction with PI value equal to 2.46 than CBL with PI value of 54.53, as shown in Figure 9. Meanwhile, THC and CBD only inhibited the SARS-CoV-2 main protease. Yet, CBD exhibited a relatively potent inhibition with a PI value of 4.43, while THC activity was only a moderate inhibitory effect with a PI value of 38.64. On the other hand, CBN, CBC, CBE, and CBF explicitly prevented the SARS-CoV-2 spike protein from interacting with the human ACE2 receptor. However, only CBF showed a strong inhibitory effect (PI value of 0.79). Meanwhile, CBC demonstrated only a potent relative impact, PI value = 4.99, and CBE and CBF exhibited a moderate activity with PI values around 35 to 39. Therefore, cannabinoid derivatives exhibited a diverse degree of specificity and potency against SARS-CoV-2 infective mechanisms (Figure 9).

Scientists have proposed a partial correlation and similarity between neuraminidase and coronavirus spike protein [54,55]. During the viral entry process, a part of the SARS-CoV-2 spike protein (namely glycan-binding domain, GBD) must also interact with mucin, a protective layer at the human lung surface [55], Figure 10B. Therefore, some neuraminidase inhibitors might also inhibit the SARS-CoV-2 spike protein GBD and disrupt its interaction with the human ACE2 receptor. In this study, the authors’ result agrees with the proposed hypothesis, demonstrating in our findings that some cannabinoid derivatives can inhibit both neuraminidase and SARS-CoV-2 spike protein interaction, as shown in Figure 9.

For example, CBG was able to inhibit not only viral but also bacterial neuraminidases with strong to relatively strong inhibitory activity (PI values around 0.6 to 9) and showed a potent relative effect against SARS-CoV-2 spike protein interaction (PI value = 2.46). On the other hand, CBE demonstrated a solid inhibition toward viral neuraminidase reaction, a PI value of 0.87, and moderately blocked SARS-CoV-2 spike protein–human ACE2 interaction with a PI value of 35.75. Finally, CBC and CBL only inhibited bacterial neuraminidase with a relatively potent inhibition, with PI values between 5 and 8. Yet, only CBC could transfer the same inhibitory potency to disrupt SARS-CoV-2 spike protein–human interaction with a PI value = 4.99. Our findings here hint at potential research and pharmaceutical revenue for developing a dual-action inhibitor against both microbial neuraminidase and SARS-CoV-2 spike protein interaction since no clinical drug is currently available.

To the best of the authors’ knowledge, the obtained results in this study demonstrate for the first time that cannabinoid derivatives can be considered as potential plant-based bioactive metabolites against *C. perfringens* and influenza A neuraminidases, and SARS-CoV-2 main protease and spike protein–human ACE2 interaction. The authors’ findings have provided the essential preliminary scientific background for further drug development from cannabis and cannabinoids. For example, developing health-related products targeting a high concentration of CBD or CBG can be beneficial since both cannabinoids are non-psychoactive substances. Nevertheless, further in vivo and clinical studies are required to confirm the authors’ in vitro findings.

### 4.3. Limitation

It is important to emphasize that the authors only performed an anti-infective screening of selected nine cannabinoids using standard in vitro biochemical enzyme-binding assays. Therefore, further investigations in cell-based, in vivo, and clinical models are needed to validate the author’s preliminary findings in this study.

### 4.4. Projection

Another aspect the authors want to discuss slightly further is that obtaining cannabinoids via oral or through smoking might alter microbiota in the human gut and lung axis, projecting from the authors’ results. The authors demonstrated three cannabinoid anti-infective potential mechanisms, as mentioned above (Figure 10A,B). First, cannabinoids may potentially prevent pathogenic bacteria like *C. perfringens* from penetrating the gut protective layer. Second, cannabinoids are likely to diminish influenza A virus replication. Third, cannabinoids may be essential in blocking SARS-CoV-2 from entering human lung tissue and disrupting SARS-CoV-2 reproduction. These discovered cannabinoids’ anti-infective potential mechanisms here are likely to contribute to maintaining and restoring microbiota homeostasis in the human gut and lungs by preventing pathogen colonization and replication. Many recent reports have proposed cannabinoids’ effects on the microbiota–gut–lung axis. Still, nearly all focused only on the impact of the endocannabinoid system contributing to inflammation [56,57], not a direct effect of cannabinoids on the pathogens. The authors’ result here may lead to a new research avenue of cannabinoids as potential bioactive molecules modulating human microbial communities in the gut–lung axis (Figure 10C). Therefore, further studies on how cannabinoid derivatives could alter gut and lung microbiota may lead to a better understanding of the relationship between bioactive metabolites derived from foods and microbiota in the human gut–lung axis.

## 5. Conclusions

In this study, the authors reported the anti-infective potential of nine selected cannabinoids against three common pathogenic mechanisms of *C. perfringens*, influenza A, and SARS-CoV-2 viruses for the first time. The results show that cannabinoids are a promising natural product class against *C. perfringens* and influenza A neuraminidases, and SARS-CoV-2 main protease and spike protein–human ACE2 interaction. Therefore, this study provides a solid scientific background for research on the pharmaceutical application of cannabinoids. However, further investigation is needed to confirm this study’s findings and reveal the true anti-infective potential of cannabinoids.

## Figures and Tables

**Table 1:** Summary of anti-infective screening activities of selected nine cannabinoids and positive control in each experiment.

No.	Compound Names	% Inhibition			
		*C. perfringen* Neuraminidase	Influenza A Viral Neuraminidase	SARS-CoV-2 Main Protease	SARS-CoV-2 Spike Protein–Human ACE2 Interaction
1	THC	99.53 (± 0.81) *	100.00 (± 0.00) *	-	57.82 (± 2.88)
2	CBD	97.24 (± 2.41) *	100.00 (± 0.00) *	-	56.30 (± 2.54)
3	CBG	100.00 (± 0.00) *	100.00 (± 0.00) *	86.99 (± 2.44) *	97.60 (± 4.16) *
4	CBN	34.87 (± 3.08)	100.00 (± 0.00) *	-	71.35 (± 8.21)
5	CBC	95.05 (± 1.04) *	ND	74.96 (± 2.25)	95.25 (± 4.54) *
6	CBL	94.25 (± 3.45) *	100.00 (± 0.00) *	100.00 (± 0.00) *	64.71 (± 4.64)
7	CBT	100.00 (± 0.00) *	27.10 (± 0.77)	62.00 (± 14.60)	53.37 (± 5.99)
8	CBF	-	13.86 (± 1.85)	65.29 (± 3.28)	99.22 (± 1.36) *
9	CBE	-	100.00 (± 0.00) *	80.26 (± 4.87)	73.66 (± 0.50)
10	Positive control	96.07 (± 2.06)	100.00 (± 0.00)	86.60 (± 23.20)	100.00 (± 0.00)

Asterisk (*) indicates positive hits, with a % inhibition of more than 80%. “ND” represents non-detectable, indicating no inhibitory activity, and “-” indicates excluding from the experiment.

**Table 2:** IC_50_ values of anti-infective screening activities of selected nine cannabinoids and positive control in each experiment.

No.	Compound Names	IC_50_ Value (µM)		
		*C. perfringen* Neuraminidase	Influenza A Viral Neuraminidase	SARS-CoV-2 Main Protease
1	THC	20.3	38.34	16.23
2	CBD	4.53 *	20.9	1.86 *
3	CBG	6.92 *	8.92 *	5.68 *
4	CBN	-	340.68	-
5	CBC	56.89	-	-
6	CBL	96.12	175.34	5.67 *
7	CBT	150.97	-	-
8	CBF	-	-	-
9	CBE	-	0.87 **	-
10	Positive control	10.99	1.00	0.42

Asterisk (*) indicates the IC_50_ value of positive hits below 10 µM, and double asterisk (**) represents the IC_50_ value of a positive hit below 1 µM. “-” indicates excluding from the experiment.

## Data Availability

The data presented in this study can be made available upon request.

## Supplementary Materials

The following supporting information can be downloaded at https://www.mdpi.com/article/10.3390/cimb47030185/s1.
