# Supplementary material for: Anti-Infective Screening of Selected Nine Cannabinoids Against Clostridium perfringens and Influenza A (H5N1) Neuraminidases, and SARS-CoV-2 Main Protease and Spike Protein Interactions
Source: Curr Issues Mol Biol. 2025 Mar 12;47(3):185. doi: 10.3390/cimb47030185 (PMC11941704; doi:10.3390/cimb47030185)
Supplement: Supplementary file 1 [file cimb-47-00185-s001.zip › cimb-3455524-supplementary.pdf]

Article

Anti-infective screening of selected nine cannabinoids against *Clostridium perfringens* and influenza A (H5N1) neuraminidases, and SARS-CoV-2 main protease and spike protein interactions

Thanet Pitakbut [1,2,*] and Oliver Kayser [2,*]

1 Pharmaceutical Biology, Department of Biology, Friedrich-Alexander-Universität Erlangen-Nürnberg (FAU), 91058 Erlangen, Germany; thanet.pitakbut@fau.de
2 Technical Biochemistry, Department of Biochemical and Chemical Engineering, TU Dortmund University, 44227 Dortmund, Germany; thanet.pitakbut@tu-dortmund.de and oliver.kayser@tu-dortmund.de.
* Correspondence: oliver.kayser@tu-dortmund.de and thanet.pitakbut@tu-dortmund.de

Supplementary file

Figure S1. Protein pairwise alignment of neuraminidase between influenza A and *C. perfringens*2

Table S1. Potency index value and calculation from antibacterial and antiviral neuraminidase experiments 3
Table S2. Potency index value and calculation from anti-SARS-CoV2 experiments 4
Table S3. Heatmap raw data 5

Figure S1. Protein pairwise alignment of neuraminidase between influenza A and *C. perfringens*

```
Alignment:   Global Protein alignment.
Parameters:  Scoring matrix:  BLOSUM 62

   Mol 1 ABU94738.1 (1 to 449)      Mol 2  WP_224383915.1 (1 to 382)
   Number of sequences to align:  2
   Total length of aligned sequences with gaps:  480 aas
   Settings:  Similarity significance value cutoff:  >= 90%


Summary of alignment results:
   Total length of aligned sequences with gaps:  480 aas
   Matching bases:    81 / 480    (16%)
   Gaps:              129 / 480    (26%)


ABU94738.1              1  -MNPNQKIITIGSICMVIGIVSLMLQIGNMISIWVSHSIQTGNQHQAEPIRNANFLTENA
WP_224383915.1          1  MYNKNN---TFEKNLDISHKPEPLILFNKDNNIWNSKYFR---------IPNIQLLNDGT

ABU94738.1             60  VASVTLAGNSSLCPVRGWAVHSKDNSIRIGSKGDVFVIREPFISCSHLECRTFFLTQG--
WP_224383915.1         49  ILTF--------------------SDIRYNGPDD-----HAYIDIASARSTDFGKTWSYD

ABU94738.1            118  -ALLNDKHSN--GTVKDRSPHRTLMSCPVGEAPSPYNSRFESVAWSASACHDGTSWLTIG
WP_224383915.1         84  IAMKNNRIDSTYSRVMDSTTVIT-NTCRILLIAGSWNTNGN---WAMTTSARRSDWSVQM

ABU94738.1            175  ISGPDNGAVAVLKYNGIITDTIKSWRNNILRTQESECACVNGSCFTVMTDGPSNGQASYK
WP_224383915.1        140  IYSDDNGL----------------TWSNKIDLTKDSSKVKNQPSNTIGWLGGVGSG-----

ABU94738.1            235  IFKMEKGKVVKSVEL----NAPNYHYEECSCYPDAGEITCVCRDNWHGSNRPWVSFNQNL
WP_224383915.1        180  -IVMDDGTIVMPAQISLRENNENNYYSLIIYSKDNGE-------TWTMGNKV--------

ABU94738.1            291  EYQIGYICSGVFGDNPRPNDGTGSCGPVSPNGAYGIKGFSFKYGNGVWIGRTKSTNSRSG
WP_224383915.1        224  -----------------PNSNTSENMVIELDGALIM---STRYDYSGYRAAYISHDLGST

ABU94738.1            351  FEMIWDP-NGWTETDSNFSVKQDIVAITDWSGYSGSFVQHPELTGLDCIRPCFWVELI--
WP_224383915.1        264  WE-IYEPLNGKVLTGKGSGCQGSFIKATTSTGHRIGLISAPKNTKGEYIRDNIAVYMIDF

ABU94738.1            408  ----RG-------RPKESTIWTSG-SSISFCGVNSDTV-----SWSWPDGAELPFTIDK-
WP_224383915.1        323  DDLSKGVQEICIPYPKDGNKLGGGYSCLSFKNGHLSIVYEANGNIEYQDLTPYYSLINKE
```

ABU94738.1 is influenza A viral neuraminidase, while WP_224383915.1 is *C. perfringens* neuraminidase.

Table S1. Potency index value and calculation from antibacterial and antiviral neuraminidase experiments

	Influenza A neuraminidase				bacterial neuraminidase			
Compound	Screen	IC50 (μM)	Potency Index	modified Potency Index	Screen	IC50 (μM)	Potency Index	Potency Index*
THC	100.00	38.34	38.34	38.34	99.53	20.3	1.85	20.30
CBD	100.00	20.98	20.98	20.98	97.24	4.53	0.41	4.53
CBG	100.00	8.92	8.92	8.92	100.00	6.92	0.63	6.92
CBN	100.00	340.68	340.68	100	34.87	ND	100	100.00
CBC	0.00	ND	100	100	95.05	56.89	5.18	56.89
CBL	100.00	175.34	175.34	100	94.25	96.12	8.75	96.12
CBT	27.10	ND	100	100	100	150.97	13.74	100.00
CBF	13.86	ND	100	100	-	-	-	
CBE	100.00	0.87	0.87	0.87	-	-	-	
Std	100.00	1	1	1	96.07	10.99	1	10.99
								Compared to std influenza A

Std = standard reference (Positive control). For antiviral neuraminidase, zanamivir (a clinical drug) is used as a reference, while for antibacterial neuraminidase, quercetin is used as a standard reference for antibacterial neuraminidase.

Table S2. Potency index value and calculation from anti-SARS-CoV2 experiments

	SARS-CoV2 main protease			SARS-CoV2 spike protein		
Compound	Screen	IC50 (μM)	Potency Index	Screen	Ratio	Potency Index
THC	-	16.23	38.64	57.82	1.73	72.94
CBD	-	1.86	4.43	56.30	1.78	77.62
CBG	86.99	5.68	13.52	97.60	1.02	2.46
CBN	-	ND	100	71.85	1.39	39.18
CBC	74.96	ND	100	95.25	1.05	4.99
CBL	100.00	5.67	13.50	64.71	1.55	54.53
CBT	62.00	ND	100	53.37	1.87	87.36
CBF	65.29	ND	100	99.22	1.01	0.79
CBE	80.26	ND	100	73.66	1.36	35.75
Std	86.60	0.42	1	100	1	1

Std = standard reference (Positive control). For antiviral main protease, GC376 (comes with an assay kit) is used as a reference, while for anti-SARS-CoV2 spike protein-human ACE2 interaction, pinostorbin is used as a standard reference.

Table S3. Heatmap raw data

	bacterial neuraminidase	Influenza A neuraminidase	SARS-CoV2 main protease	SARS-CoV2 spike protein
Compounds	Potency Index	Potency Index	Potency Index*	Potency Index
THC	1.85	38.34	38.64	72.94
CBD	0.41	20.98	4.43	77.62
CBG	0.63	8.92	13.52	2.46
CBN	100	100	100.00	39.18
CBC	5.18	100	100.00	4.99
CBL	8.75	100	13.50	54.53
CBT	13.74	100	100.00	87.36
CBF	-	100	100.00	0.79
CBE	-	0.87	100.00	35.75

	0
	100
